# Supplementary material for: Genome-Assisted Probiotic Characterization and Application of Lactiplantibacillus plantarum 18 as a Candidate Probiotic for Laying Hen Production
Source: Microorganisms. 2023 Sep 22;11(10):2373. doi: 10.3390/microorganisms11102373 (PMC10609342; doi:10.3390/microorganisms11102373)
Supplement: Supplementary file 1 [file microorganisms-11-02373-s001.zip › microorganisms-2591280-supplementary.pdf]

Supplementary Table S1. Dietary composition and nutrient levels of the experimental diets (as fed basis)

Ingredients	%	Nutrient content [c]	Value
Corn	58.98	Metabolizable energy (MC/kg)	2.60
Soybean meal (43)	18.22	Crude protein (%)	15.50
Limestone powder (10-20 mesh)	9.73	Crude fibre (%)	2.94
DDGS	8.47	Calcium (%)	3.70
Rice bran meal (15.1%)	2.00	Total phosphorus (%)	0.47
Corn germ cake (16.7%)	0.79	Salt (%)	0.36
Rice bran oil	0.60	K (%)	0.62
Bone Calcium hydrogen phosphate	0.50	Na (%)	0.20
NaCl	0.25	Cl (%)	0.2
Methionine	0.12	Lysine (%)	0.75
Lysine sulfate (70%)	0.10	Methionine (%)	0.37
mineral premix [a]	0.10	*DL*-Methionine (%)	0.63
Choline chloride (50%)	0.10	Threonine (%)	0.58
Multi-vitamins [b]	0.03	Tryptophan (%)	0.16
Thermostable phytase (20000)	0.01	Isoleucine (%)	0.60
Total	100.00	Valine (%)	0.72

[a] Mineral premix provided the following per kg of diets: Cu,7,000mg; Fe ,78,000mg; Zn, 65,800mg; Mn ,85,000; I ,550mg; Se, 300mg.

[b] Multi-vitamins provided the following per kg of diets: VA, 4,000 IU; VD3, 1,500 IU; VK3, 15,000 mg; VE, 95,000 IU; VB1,10,000 mg; VB2, 30,000mg; VB6, 15,000 mg; VB12, 120 mg; Nicotinamide, 50,000mg; D biotin, 500mg; folic acid, 800 mg; D pantothenic acid, 45,000; Choline, 500 mg.

[c] The values of metabolizable energy, available phosphorus, and amino acids are calculated, and others are measured values.

DDGS: Distillers Dried Grains with Solubles

Supplementary Table S2. Genes associated with plantaricin in the LP18 genome

query	Description	Preferred_name
CHROMOSOME_1_2585	LytTr DNA-binding domain	plnD
CHROMOSOME_1_2587	-	plnF
CHROMOSOME_1_2592	CAAX protease self-immunity	plnU

Supplementary Table S3. Genes associated with antibiotic resistance in the LP18 genome of the CARD database

Contig	NZ_CP016270.1_52	NZ_CP016270.1_1972
Start	53399	2061681
Stop	54136	2061989
Orientation	+	-
Cut_Off	Strict	Strict
Pass_Bitscore	50	75
Best_Hit_Bitscore	86.7	75.1
Best_Hit_ARO	vanY gene in vanB cluster	qacJ
Best_Identities	31.33	40.2
ARO	3002956	3007014
Model_type	protein homolog model	protein homolog model
SNPs_in_Best_Hit_ARO	n/a	n/a
Other_SNPs	n/a	n/a
Drug Class	glycopeptide antibiotic	disinfecting agents and antiseptics
Resistance Mechanism	antibiotic target alteration	antibiotic efflux
AMR Gene Family	vanY; glycopeptide resistance gene cluster	small multidrug resistance (SMR) antibiotic efflux pump
Percentage Length of Reference Sequence	91.42	95.33
ID	gnl|BL_ORD_ID|603|hsp_num:0	gnl|BL_ORD_ID|4661|hsp_num:0
Model_ID	556	5730

Supplementary Table S4. Genes associated with virulence factors in the LP18 genome of the VFDB database

**VFclass**	**Virulence factors**	**Related genes**	**Lactiplantibacillus plantarum 18(Prediction)**
			**NZ_CP016270.1**
Adherence	BslA	bslA	-
	Listeria adhesion protein(Listeria)	lap	orf02247
	Streptococcal plasmin receptor/GAPDH(Streptococcus)	plr/gapA	orf02888
Enzyme	Immune inhibitor A metalloproteinase	Undetermined	-
		inhA	-
	Pho	plcA	-
	Phosphatidylinositol-specific phospholipase C (PI-PLC)	piplc	-
	Sphingomyelinase (SMase)	sph	-
	Streptococcal enolase(Streptococcus)	eno	orf00802
Immune evasion	B. cereus exo-polysaccharide (BPS)	bpsA、bpsB、bpsC、bpsD、bpsE、bpsF、bpsG、bpsH、bpsX	-
	Hyaluronic acid (HA) capsule	hasA、hasB、hasC	-
	Polyglutamic acid capsule	capA、capB、capC、capD、capE	-
	Capsule(Streptococcus)	cps4I	orf00192
		cps2T	orf00201
		rmlA	orf00206; orf00222
		rmlB	orf00208
		rfbD	orf00209
		epsE	orf00215
		cpsY	orf00747
	Polysaccharide capsule	Undetermined	orf02158; orf02819; orf02860
Iron acquisition	Bacillibactin	dhbA、dhbB、dhbC、dhbE、dhbF	-
	Hal	hal	-
	IlsA	ilsA	-
		asbA、asbB、asbC、asbD、asbE、asbF	-
Regulation	AcpAB	acpA、acpB	-
	AtxA	atxA	-
	PagR-XO1	pagR-XO1	-

	PagR-XO2	pagR-XO2	-
	PlcR-PapR quorum sensing	papR、plcR	-
	LisR/LisK(Listeria)	lisR	orf00468
Secretion system	Type VII secretion system	essC、esxB、esxL	-
	T6SS-II(Klebsiella)	clpB	orf00786
Toxin	Anthrax toxin	Cya、Lef、pagA	-
	Anthrolysin O/Cereolysin O/Hemolysin I	alo	-
	Cereulide	cesA、cesB、cesC、cesD、cesH、cesP、cesT	-
	Certhrax	cer	-
	Cytotoxin K (Hemolysin IV)	cytK	-
	Hemolysin II	hlyII	-
	Hemolysin III homolog	Undetermined	-
	Hemolysin III	hlyIII	-
	Hemolytic enterotoxin HBL	hblA、hblC、hblD	-
	Insecticidal crystalline toxins	Cry、Cyt、vip	-
	Non-hemolytic enterotoxin (Nhe)	nheA、nheB、nheC	-
	Phytotoxin phaseolotoxin(Pseudomonas)	cysC1	orf00351
	Hemolysin(Clostridium)		orf00522
Antiphagocytosis	Capsule(Enterococcus) Capsule(Klebsiella)	cpsI	orf00227
		gnd	orf00466
			orf00916
		cpsJ	orf02568
Bile resistance	Bile-salt hydrolase(Listeria)	bsh	orf02198
Invasion	Cell wall teichoic acid glycosylation protein(Listeria)	gtcA	orf01556
Manganese uptake	Pneumococcal surface antigen A / Metal binding protein SloC(Streptococcus)	psaA	orf00123
Phagosome arresting	Nucleoside diphosphate kinase(Mycobacterium)	ndk	orf02475
Protease	Trigger factor(Streptococcus)	tig/ropA	orf01024
	Serine protease(Streptococcus)	htrA/degP	orf02301
Stress adaptation	Catalase(Neisseria)	katA	orf02226
Surface protein anchoring	Lipoprotein diacylglyceryl transferase(Listeria)	lgt	orf02858
Table saved from VFDB (http://www.mgc.ac.cn/VFs/) [Fri Jul 7 11:13:13 2023]			

Supplementary Figure S1. Prediction of CRISPR structural positions in the LP18 genome

CRISPR ID : CHROMOSOME_1_1

- Left flanking sequence: (100 bp [start=2455201;end=2455300], AT%=70): TCGAATGGAAAAGTTCAAAAATAGTTGTTATTATTATGTTGACGAGGATTTAGTAGACTGGCGTGATATGAATTAATGAGTAAATGATGTTAAAATGCTG
- CRISPR start position: 2455301 ---------- CRISPR end position: 2455865 ---------- CRISPR length: 564
- DR consensus: GTCTTGAATAGTAGTCATATCAAACAGGTTTAGAAC
- DR length: 36 Number of spacers: 8
- Consensus Repeat ID (Nb in CRISPRdb): Unknown (0)
- CRISPRDirection: ND
- Potential orientation (AT%): +
- Conservation of DRs based on Entropy: 100
- Conservation of Spacers: 0
- Evidence Level: 4

2455301	GTCTTGAATAGTAGTCATATCAAACAGGTTTAGAAC	TTGAAGTCCCAGCCGTGAACCAGTTAATGA	2455366
2455367	GTCTTGAATAGTAGTCATATCAAACAGGTTTAGAAC	TCAAATTAAGAATGCCGTAGCCACAATTAC	2455432
2455433	GTCTTGAATAGTAGTCATATCAAACAGGTTTAGAAC	GGACGCATCGGGGTATATTGCTGACGAAGA	2455498
2455499	GTCTTGAATAGTAGTCATATCAAACAGGTTTAGAAC	AGAACGGTACGTATGAGTTAACACCTAAAT	2455564
2455565	GTCTTGAATAGTAGTCATATCAAACAGGTTTAGAAC	TTGTATTATCTAATCGTAATTGAGTACCGTA	2455631
2455632	GTCTTGAATAGTAGTCATATCAAACAGGTTTAGAAC	GCTATACTATAAACATATATAAGGAAAGGA	2455697
2455698	GTCTTGAATAGTAGTCATATCAAACAGGTTTAGAAC	GATTAAATGCAAGTACTGTCTTTCGAATAT	2455763
2455764	GTCTTGAATAGTAGTCATATCAAACAGGTTTAGAAC	ACGTCGGCGAAATCTTCACCACGCCGATTA	2455829
2455830	GTCTTGAATAGTAGTCATATCAAACAGGTTTAGAAC		2455865

- Right flanking sequence: (100 bp [start=2455866;end=2455965], AT%=60): GATGCTGAGGAAACGAAGCATAGAACGTTTGTCTTGAATAGCGGCCACATCAAAAACAGTTTCACGATACTAAAAAATGGCTCACCAACTAAAACAGGTT
